# The key role of ubiquitination and sumoylation in signaling and cancer: a research topic

**DOI:** 10.3389/fonc.2012.00187

**Published:** 2012-12-05

**Authors:** Wenyi Wei, Hui-Kuan Lin

**Affiliations:** ^1^Department of Pathology, Beth Israel Deaconess Medical Center, Harvard Medical SchoolBoston, MA, USA; ^2^Department of Molecular and Cellular Oncology, M. D. Anderson Cancer Center, The University of TexasHouston, TX, USA; ^3^Graduate School of Biomedical Sciences, The University of TexasHouston, TX, USA

## Introduction

Ubiquitination and sumoylation are two important post-translational modifications that play pivotal roles in signaling regulation, protein trafficking, protein stability, and transcriptional regulation, ultimately regulating a plethora of biological processes such as cell survival, cell migration, DNA damage response (DDR), neurodegeneration, and cancer. Although ubiquitination is traditionally viewed as a critical mark targeting proteins for proteasome-dependent degradation, recent studies have revealed that it also has non-proteolytic functions. In contrast, sumoylation is long thought not to target proteins for degradation, but accumulating evidence suggests that it can serve as a priming pre-requisite for ubiquitination, thereby inducing protein ubiquitination and degradation. Thus, there is an important cross-talk between sumoylation and ubiquitination in determining protein fate. Deregulation in these two processes may cause aberrant activity of proteins and in turn contributes to cancer development. In this Research Topic, we assembled 10 review articles to discuss the role of these two post-translational modifications in regulating diverse signal transduction pathways, thereby providing novel insights in unraveling the puzzle as to how they may regulate cancer progression.

The ubiquitin-proteasome system has been recently characterized as a major regulatory mechanism for ensuring ordered and coordinated cell cycle progression by selective degradation of key cell cycle regulators. Two related, multi-subunit E3 ubiquitin ligase enzymes, the Anaphase Promoting Complex (APC) and the Skp1-Cullin1-F-box complex (SCF) are thought to be the major driving forces governing cell cycle progression. Nagi Ayad's group discussed the important physiological functions of APC/C as well as the upstream signaling pathway that governs the timely regulation of APC/C. Given the critical role of APC/C in cell proliferation and development, they also illustrated the emerging contribution of APC/C in tumorigenesis and proposed APC/C intervention as a potential anti-cancer therapeutic approach.

Among the SCF-type of E3 ubiquitin ligases, SCF^Skp2^ is one of the most well-studied E3 ligases and Skp2 overexpression is frequently observed in various types of human cancers including breast cancer. The Wei group recently summarized the oncogenic role of Skp2 in breast cancer development. The interplay between Skp2 and other major signaling pathways as well as the recent advances in identifying Skp2 downstream substrates were also discussed. Lastly, they proposed specific Skp2 inhibitors as novel anti-breast cancer agents.

Unlike Skp2, VHL forms a distinct type of E3 ligase by associating with Elongin B, Elongin C, and Cullin 2. The Yang group discussed the important role of VHL in hypoxia sensing and kidney disease development by targeting HIF for ubiquitination and destruction in a hydroxylation-dependent manner. They also summarized the recent advances in understanding the tumor suppressor role of VHL independent of HIF. The identification of novel VHL substrates including PKC and EGFR and their relevance to signaling and cancer development were also discussed.

In addition to associating with VHL, the Elongin B/C complex can also interact with the SOCS box-containing proteins and Cullin 2 or Cullin 5 to form distinct functional E3 ubiquitin ligases. The Takumi Kamura group contributed a comprehensive review that described the recent advances in further characterizing the assembly as well as the physiological functions of the Elongin B/C-containing E3 ligases, which are further divided into the Cullin 2-type and the Cullin 5-type. They also summarized the newly identified downstream substrates, which provided an important insight into the critical roles of Elongin B/C-containing E3 ligases in regulating a variety of cellular functions.

The Pengbo Zhou group summarized the recent progress in functional analysis of another major class of E3 ubiqutin ligases, the Cullin 4-containing (CRL4) family of ligases. In this review, the authors updated the recent understanding of the two Cullin 4 family members, Cullin 4A and Cullin 4B in human cancer and neuronal disease development. In addition, the recent advances in identifying novel substrates for various Cullin 4-containing E3 ligases that typically associate with a specific DDB1-Cullin 4 associated factor (DCAF) were further discussed. More importantly, given their critical roles in tumorigenesis, the authors speculated that Cullin 4A and Cullin 4B could be potentially pursued as new targets for cancer prevention.

Protein kinases such as Akt, MAPK, and IKK are commonly upregulated and/or activated in a variety of human cancers. The Hung group summarized recent advances of how these oncogenic kinases regulate protein ubiquitination and degradation. They proposed that protein phosphorylation induced by these kinases provides a scaffold for the recognition of ubiquitin ligases (E3s), thereby eliciting protein ubiquitination and degradation. Several oncogenic proteins, such as β-catenin, MCL-1, and Snail, known to be regulated by these oncogenic kinases, were also discussed. In addition to kinase regulation, ubiquitination can also orchestrate DDR by controlling protein degradation and non-proteolytic pathways. DDR triggers serial signaling events that are involved in DNA damage repair, transcription regulation, growth arrest, and apoptosis. The Jin group discussed the role of Cullin/RING ubiquitination ligase 1 (CRL1) and CRL4 in multiple steps of the DDR by regulating protein stability. The Yan group further illustrated the role of histone ubiquitination and deubiquitination in regulating transcription and DDR. The E3 ligases and deubiquitinating enzymes regulating the histone ubiquitination and deubiquitination processes and DDR are discussed.

Like protein ubiquitination and phosphorylation, sumoylation is another important post-translational modification that is also critically involved in DDR. Upon DNA damage, sumoylation of proteins like 53BP1 and BRCA-1 involved in DDR and DNA repair is induced and may play a role in DDR by inducing its E3 ligase activity. David Ann and colleagues discussed the potential crosstalk between sumoylation and ubiquitination and speculated that RNF4 may be a critical E3 ligase that connects the sumoylation and ubiquitination crosstalk to participate in ATM-mediated signaling and DDR.

Although ubiquitination is traditionally viewed as a critical marker targeting proteins for proteasome-dependent degradation, recent studies reveal that it also plays non-proteolytic functions. K48-linked ubiquitination in general leads to protein degradation, while K63-linked ubiquitination fails to do so. The Lin group and colleagues focused on a new type of ubiquitination called K63-linked ubiquitination and proposed that K63-linked ubiquitination serves as an important activation mark for oncogenic kinase activation, thereby contributing to cancer development. Thus, targeting K63-linked ubiquitination may be employed for cancer therapy.

